#  Intrahepatic Cholangiocarcinoma: Clinical Aspects, Pathology and Treatment

**DOI:** 10.1155/1992/93976

**Published:** 1992

**Authors:** Richard T. Schlinkert, David M. Nagorney, Jon A. van Heerden, Martin A. Adson

**Affiliations:** Department of Surgery Mayo Clinic 200 First Street, SW Rochester MN 55905 USA

## Abstract

Intrahepatic cholangiocarcinoma (ICC) is the second most common primary tumor of the liver. To further define its clinicopathology and surgical management, we reviewed our experience. Clinical presentations of 32 patients with ICC was similar to that with hepatocellular carcinoma. Jaundice occurred in only 27 percent. ICC was unresectable due to advanced disease stage in 81 percent. Six patients had curative resections with two 5 year disease free survivors. Underlying liver disease was associated with ICC in 34 percent of patients.


*HPB Surgery*, 1992, Vol. 5, pp. 95–102
Reprints available directly from the publisher
Photocopying permitted by license only

© Harwood Academic Publishers GmbH
Printed in the United Kingdom

### INTRAHEPATIC CHOLANGIOCARCINOMA: Clinical Aspects, Pathology and Treatment

RICHARD T. SCHLINKERT, DAVID M. NAGORNEY, JON A. VAN HEERDEN and MARTIN A. ADSON

*Department of Surgery, Mayo Clinic, Rochester, Minnesota, USA*

*(Received 7 January 1991)*

Intrahepatic cholangiocarcinoma (ICC) is the second most common primary tumor of the liver. To further define its clinicopathology and surgical management, we reviewed our experience. Clinical presentations of 32 patients with ICC was similar to that with hepatocellular carcinoma. Jaundice occurred in only 27 percent. ICC was unresectable due to advanced disease stage in 81 percent. Six patients had curative resections with two 5 year disease free survivors. Underlying liver disease was associated with ICC in 34 percent of patients.

KEY WORDS: Intrahepatic cholangiocarcinoma

### INTRODUCTION

Cholangiocarcinoma of the intrahepatic bile ducts (peripheral cholangiocarcinoma) is the second most common primary malignancy arising within the liver[1]. This tumor, however, accounts for less than 10% of primary hepatic malignancies[2,3]. Because of its relative rarity, little is known about either the clinicopathologic features of this tumor or the results of therapy[4,5]. To further define the clinical presentation and surgical management of this histologic subtype of primary hepatic tumor, we reviewed the surgical management of patients with intrahepatic cholangiocarcinoma treated at our institution.

### *Patients and Methods*

Thirty two patients with intrahepatic cholangiocarcinoma (ICC) were managed at the Mayo Clinic between 1965 and 1980. Only those patients who had their initial diagnosis and treatment at the Mayo Clinic were included in this review. ICC was defined as a malignancy of intrahepatic bile duct origin presenting as a focal liver mass. Patients with cholangiocarcinoma arising from the bile duct confluence, extrahepatic bile duct, and the gallbladder, with or without local infiltration of the liver, were excluded. A concurrent diagnosis of cirrhosis was based on the histologic findings of diffuse bridging fibrosis and regenerative nodules regardless of primary liver disease.

Patient demographics, clinical features, laboratory findings, operative management, and hospital morbidity and mortality were recorded for all patients.

Address correspondence to: David M. Nagorney, M.D., Department of Surgery, Mayo Clinic, 200 First Street, SW, Rochester, MN 55905, USA

Followup data were obtained for all patients by telephone interview, correspondence, or review of the patients' current medical records and extended from the date of diagnosis to death or July 1989. All surviving patients have been followed for a minimum of five years. Survival was determined by the Kaplan-Meier method using the Statistical Analysis System (SAS) software package and was actual because of the study period. No patient was lost to follow-up.

### RESULTS

### *Demographics*

There were 17 women and 15 men with a mean age of 55 years and a range of 31 to 81 years. Associated hepatobiliary and gastrointestinal diseases were not uncommon (Table 1). Seven patients had cirrhosis and two additional patients had clinical cirrhosis evident by the physical stigmata of chronic liver disease and varices, though unconfirmed histologically. Two other patients had diffuse intrahepatic primary sclerosing cholangitis, and five patients had inflammatory bowel disease.

One patient had past exposure to Thorotrast. Forty-six percent of patients had a family history of carcinoma, though extrahepatic in origin. Sixteen patients had had a prior cholecystectomy, 15 of whom had a cholecystectomy longer than six months prior to the diagnosis of cholangiocarcinoma. Chronic exposure to tobacco was present in 51% of patients: cigarettes in 34%, and cigar, pipe tobacco, or oral use in 17%. Five women had taken oral contraceptives. Alcohol abuse was documented in 6%.

### *Signs and Symptoms*

The clinical features of the patients are listed in Table 2. Abdominal pain or discomfort, malaise, and weight loss were the primary presenting symptoms. Hepatomegaly or an abdominal mass was the main physical finding. In contrast to patients with extrahepatic cholangiocarcinoma, jaundice occurred in only 27% of patients.

**Table 1** Chronic hepatobiliary diseases in 32 patients with intrahepatic cholangiocarcinoma

| *Disease* | *Number of Patients* | % |
|---|---|---|
| Cirrhosis (biopsy proven) | 7 | 22 |
| Hemochromatosis | 4 | |
| Secondary biliary | 2 | |
| Unspecified | 1 | |
| Sclerosing cholangitis | 2 | 6 |
| Inflammatory bowel disease | 5 | 16 |
| Chronic ulcerative colitis | 3 | |
| Crohn's disease | 1 | |
| Nonspecific colitis | 1 | |

**Table 2** Clinical features of 32 patients with intrahepatic cholangiocarcinoma

| *Symptoms* | *Frequency (%)* |
|---|---|
| Pain | 68 |
| Fatigue | 35 |
| Weight loss | 26 |
| Anorexia | 26 |
| Pruritus | 19 |
| Fever | 19 |
| Jaundice | 13 |
| Nausea | 11 |
| Other gastrointestinal (bloating, early satiety, pressure) | 19 |
| Trhombophlebitis | 3 |
| *Signs* | |
| Hepatomegaly | 60 |
| Nodular | 33 |
| Smooth | 27 |
| Mass | 30 |
| Jaundice | 27 |
| Ascites | 17 |
| Splenomegaly | 10 |
| Tender right upper quadrant | 9 |
| Stigmata chronic liver disease | 9 |

### *Pathology and Extent of Disease*

Histologically, 91% of patients had a pure cholangiocarcinoma and 9% had a mixed cholangiohepatocellular carcinoma but with cholangiocarcinoma predominance. Ten percent of tumors were well-differentiated grade I, 50% were grade II, and 40% grades III-IV. Tumors were solitary in 60% of patients and multicentric in 40%. Seventy-one percent of patients had bilobar hepatic involvement. The site of origin of solitary cholangiocarcinomas or the dominant mass of multicentric cholangiocarcinoma was distributed equally between the hepatic lobes. Of the 24 patients who had surgery exploration, four patients had documented regional nodal metastases. Distant metastases to peritoneum, lung, and bone, respectively, were documented in three patients.

### *Surgical Treatment*

Eight patients (25%) had cholangiocarcinomas biopsied either percutaneously or at peritoneoscopy. Bilobar multicentricity obviated exploration for resection. Seventeen patients (53%) had exploratory celiotomy and biopsy only. Tumors in these patients were judged unresectable because of major vascular invasion, multicentricity, or extrahepatic lymph node metastases. Hepatic lobectomy in four patients (12%) and wedge resection in two patients (6%) were performed with curative intent. One patient underwent a palliative right hepatic lobectomy for a symptomatic tumor with microscopic involvement at the margin of resection (Table 3).

**Table 3**

| *Surgical Procedure* | *Number* | % |
|---|---|---|
| None | 8 | 25 |
| Exploration with biopsy | 17 | 53 |
| Wedge resection for cure | 2 | 6 |
| Lobectomy for cure | 4 | 13 |
| Incomplete resection | 1 | 3 |

### *Survival*

Overall median survival was 5.1 months. The median survival for patients with curative resection was 2.8 years (range 9.6 months to 7.4 years) with two patients alive and disease free at 6.2 and 8.5 years (see Figure 1). The patient undergoing palliative resection died from disease 3 years postoperatively. The median survival of the 25 patients with unresectable tumors assessed intraoperatively was 3.6 months (range one week to 7.4 years). Interestingly, one 34-year-old woman had spontaneous calcification of a multicentric tumor without evidence of distant tumor progression and remains a long-term survivor in this group.

To determine whether survival was affected by factors other than resection, the relationship of survival to tumor multicentricity, bilaterality, and histologic differentiation was analysed. The median survival of solitary unilobar, unresected lesions (N = 4) of two months was not different than the median survival of three months

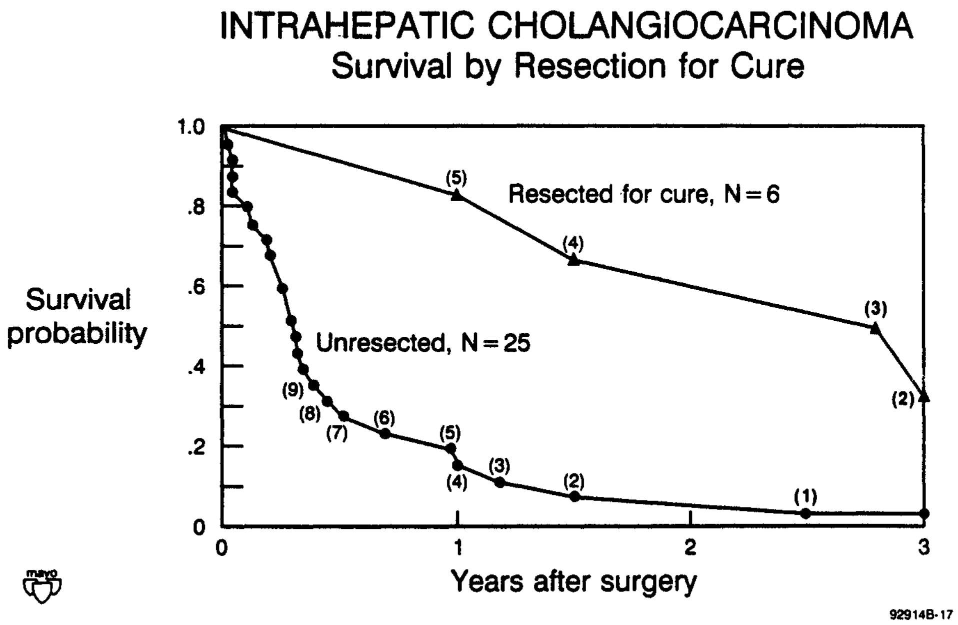

**Figure 1**

for multicentric bilateral tumors (N = 10). The median survival of unresected bilobar solitary tumors (N = 8) was 12 months. Overall median survival for grade I, II, and III tumors was 12, 8, and 8 months, respectively. Resected grade I, II, and III tumors had a median survival of 4.9 years, 2.9 years, and 2 years, respectively, whereas unresected lesions for the same categories were 12 months, 4 months, and 4 months, respectively. Palliative external beam irradiation in five patients and adjunctive chemotherapy in 16 patients had no objective influence on survival based on demonstrable disease progression after initiation of such therapy.

### *Morbidity/Mortality*

Of the 24 patients who underwent exploration, one postoperative death (4%) occurred from advanced disease following biopsy alone. Four patients (16%) had minor complications after exploration and biopsy: wound infection, lower extremity deep vein thrombosis, transient biliary fistula, and prolonged ileus respectively. One major complication occurred following curative resection which consisted of cholangitis from an obstructed T-tube which resolved with parenteral antibiotics and T-tube irrigation.

### DISCUSSION

This study demonstrates that intrahepatic cholangiocarcinoma presents clinically in a similar fashion to primary hepatocellular carcinoma but in a distinctly different manner than that of extrahepatic cholangiocarcinoma. The diagnosis is often delayed until the disease has reached an advanced stage. Although a low rate of resectability (18.7%) is the consequence of advanced disease stage, surgical resection remains the only therapeutic modality which may afford prolonged survival. The association with additional hepatobiliary pathology and inflammatory bowel disease is noteworthy but whether significant etiologic relationships exist remains unproven.

The presentation of intrahepatic cholangiocarcinoma is similar to that of other intrahepatic malignancies[2,3,4,5]. Abdominal pain and fullness, anorexia, and malaise are common findings. In contrast extrahepatic cholangiocarcinoma presents most commonly with obstructive jaundice[6,7,8]. Jaundice in patients with intrahepatic cholangiocarcinoma occurs at an advanced stage with extrinsic compression of the hepatic duct confluence by tumor, diffuse liver replacement by tumor, or extrahepatic duct occlusion by tumor emboli. While most extrahepatic cholangiocarcinomas are solitary and localized, our study suggests that intrahepatic cholangiocarcinomas are frequently multicentric and diffuse. Clearly, this pathologic feature makes surgical intervention for cure less likely and palliation difficult. Despite extensive intrahepatic involvement, only 30% of our patients undergoing laparotomy had proven metastatic disease. The propensity for both intra- and extrahepatic cholangiocarcinoma to metastatize infrequently despite locally advanced primary disease represents a pathologic characteristic of this cell type[7,8].

Worldwide, most patients with hepatocellular carcinomas have underlying cirrhosis. Although many chronic liver diseases have been well recognized as a predisposing factor for carcinomas arising from hepatocytes, similar findings have not been well documented for patients with intrahepatic cholangiocarcinoma.

Clinical evidence strongly supports a relationship between cholangiocarcinoma of either intra- or extrahepatic origin and sclerosing cholangitis. Ritchie *et al.*[9] first demonstrated an association between bile duct carcinoma and ulcerative colitis. Subsequent studies have suggested that underlying sclerosing cholangitis, which is frequently associated with ulcerative colitis, may predispose the bile duct epithelium to malignant transformation[10,11]. Recent reports[12,13] support the postulate that the incidence of cholangiocarcinoma is increased in patients with sclerosing cholangitis. Two patients had sclerosing cholangitis and five had inflammatory bowel disease in this study. An association between intrahepatic cholangiocarcinoma and cirrhosis is less firm. Edmonson and Steiner[1] confirmed cirrhosis in 3 of 13 patients (29%) with intrahepatic cholangiocarcinoma, but Kawarada *et al.*[5] found no evidence of cirrhosis in 11 patients. Our finding of cirrhosis in 23% of patients with intrahepatic cholangiocarcinoma suggest a potential association.

Our patients with intrahepatic cholangiocarcinoma presented with advanced disease and thus, our resectability rate was low. Long-term survival was poor regardless of method of treatment, but survival after diagnosis was prolonged only for those patients who underwent resection. Two patients are without evidence of disease after 5 years. Chen *et al.*[14] and Kawarada *et al.*[5] have reported similar median survival of 21 months and 18 months, respectively, after curative resection. Although our study population was small, tumor grade was the only clinicopathologic factor associated with survival. In contrast to our findings and others,[5,14] Iwatsuki and Starzl[15] have not found long-term survival affected adversely by this histologic subtype of primary hepatic malignancy. Despite the poor prognosis of patients with intrahepatic cholangiocarcinoma, most surgeons concur that hepatic resection offers the only chance for cure and at present should be pursued actively and judiciously. Hepatic transplantation may have a role for multicentric tumors unresectable by conventional hepatic resection. Indeed, given the high frequency in intrahepatic multicentricity and the relatively low frequency of extrahepatic metastases, hepatic transplantation is the most attractive alternative. To date, survival after transplantation for malignancy remains poor[16]. However it is difficult to determine whether transplantation was performed for intrahepatic or extrahepatic cholangiocarcinomas. Alternatively, Okamoto[17] has evaluated hepatic artery ligation in patients with unresectable tumors and found that this modality offered a slight but not significant improvement in survival in patients with primary hepatic malignancies compared to those who underwent noncurative resections.

### *Summary*

Intrahepatic cholangiocarcinoma presents insidiously and often late in its course. Its presentation is closely different from that of extrahepatic cholangiocarcinoma but quite similar to the presentation of intrahepatic hepatocellular carcinoma. There appears to be an association with underlying hepatobiliary pathology and while surgical cures are rare, resection appears to offer the only chance for cure in these patients and should be pursued actively. Because of late diagnosis and advanced disease at the time of presentation, our current energies should be directed towards earlier diagnosis and perhaps the identification of effective adjuvant chemo and/or radiotherapy following resection.

*References*

1. Edmonson, H.A. and Steiner, P.E. (1954) Primary carcinoma of the liver: A study of 100 cases among 48,900 necropsies. *Cancer*, **7**, 462–503
2. The Liver Cancer Study Group of Japan (1984) Primary liver cancer in Japan. *Cancer*, **54**, 1747–1755
3. Nagorney, D.M., van Heerden, J.A.,, Ilstrum, D.M. and Adson, M.A. (1989) Primary hepatic malignancy: Surgical management and determinants of survival. *Surgery*, **106**, 740–749
4. Okuda, K., Kubo, Y., Ikazaki, N., Arishnma, T., Hashimoto, M., Jinnouchi, S., Sawa, Y., Shimokawa, Y., Nakajima, Y., Noguchi, T., Nakano, M., Kojiro, M. and Nakashima, T. (1977) Clinical aspects of intrahepatic bile duct carcinoma including hilar carcinoma: A study of 57 autopsy-proven cases. *Cancer*, **39**, 232–246
5. Kawarada, Y. and Mizumoto, R. (1984) Cholangiocellular carcinoma of the liver. *The Am. J. Surg.*, **147**, 354–359
6. Beazley, R.M., Hadjis, N., Benjamin, I.S. and Blumgart, L.H. (1984) Clinicopathologic aspects of high bile duct cancer: Experience with resection and bypass surgical treatments. *Ann. Surg.*, **199**, 623–636
7. Weinbren, K. and Mutum, S.S. (1983) Pathological aspects of cholangiocarcinoma. *Journal of Pathology*, **139**, 217–238
8. Adson, M.A. and Farnell, M.B. (1981) Hepatobiliary cancer – surgical considerations. *Mayo Clinic Proceedings*, **56**, 686–699
9. Ritchie, J.K., Allan, R.N., Macartney, J., Thompson, H., Hawley, P.R. and Cooke, W.T. (1974) Biliary tract carcinoma associated with ulcerative colitis. *Quarterly Journal of Medicine*, **170**, 263–279
10. Akwari, O.E., van Heerden, J.A., Foulk, W.T. and Baggenstoss, A.H. (1975) Cancer of the bile ducts associated with ulcerative colitis. *Ann. Surg.*, **181**, 303–309
11. Wee, A., Ludwig, J., Coffey, Jr. R.J., LaRusso, N.F. and Wiesner, R.H. (1985) Hepatobiliary carcinoma associated with primary sclerosing cholangitis and chronic ulcerative colitis. *Human Pathology*, **16**, 719–726
12. Marsh, J.W., Iwatsuki, S., Makowka, L., Esquivel, C.O., Gordon, R.D., Todo,S., Tzakis, A., Miller, C., Van Thiel, D. and Starzl, T.E.(1988) Orthotopic liver transplantation for primary sclerosing cholangitis. *Ann. Surg.*, **207**, 21–25
13. Rosen, C.B., Nagorney, D.M., Wiesner, R.H., Coffey, Jr. R.J., and LaRusso, N.F. (In press) Cholangiocarcinoma complicating primary sclerosing cholangitis. *Ann. Surg.*
14. Chen, M.F., Jan, Y.Y., Wang, C.S., Jeng, L.B. and Hwang, T.L. (1989) Clinical experience in 20 hepatic resections for peripheral cholangiocarcinoma. *Cancer*, **64**, 2226–2232
15. Iwatsuki, S., Starzl, T.E. (1989) Resulting primary hepatic malignancy. *SCNA*, **69**, 315–322
16. Ringe, B., Wittekind, C., Bechstein, W.O., Bunzendahl, H. and Pichlmayr, R. (1989) The role of liver transplantation in hepatobiliary malignancy. A retrospective analysis of 95 patients with particular reference to tumor stage and recurrence. *Ann. Surg.*, **209**, 88–98
17. Okamoto, E., Tanaka, N., Yamanaka, N. and Toyosaka, A. (1984) Results of surgical treatments of primary hepatocellular carcinoma: Some aspects to improve long-term survival. *World J. Surg.*, **8**, 360--36

*(Accepted by S. Bengmark 25 March 1991)*

### INVITED COMMENTARY

Worldwide, hepatocellular carcinoma (HCC) is by far the most common histological type of primary liver cancer. In contrast, clinical experience with the cholangiocellular form is limited. Regarding surgical therapy and prognosis the extrahepatic or hilar type must be clearly differentiated from the intrahepatic or peripheral cholangiocarcinoma (CCC).

In their review on clinical aspects, pathology, and treatment of cholangiocellular carcinoma over a 15-year period Schlinkert *et al.* emphasize again that the general prognosis of those patients is poor. The median survival of 25 patients with non-resectable tumors was only 3.6 months. Due to advanced tumor stages the resectability rate was very low (18.7%) but patients after curative resection showed an improved median survival of 2.8 years, with 2 patients alive and disease free at 6.2 and 8.5 years, respectively. It is highly likely, that those prolonged survival times are not only due to the possible earlier stage of resectable patients but mainly due to surgical removal of the tumor. These both essential findings, that first the resectability rate in CCC is extremely low and second that if anything, surgery plays the main role in the therapeutic success are very much in accordance with our observations.

The question whether local resectability can be extended by total hepatectomy and subsequent liver transplantation in cases with advanced tumors has stimulated many controversial discussions, but remains open. To date, we ourselves have performed liver transplantation in 16 patients with intrahepatic cholangiocellular carcinoma, including 3 with a mixed hepatocholangiocellular type of tumor. All lesions were not resectable by conventional means; ;11 of 16 patients had advanced tumor stages IV A or IV B according to the TNM classification. Despite the fact that in half of the cases regional lymph nodes or distant metastases were not present at the time of operation all patients surviving the procedure developed early tumor recurrence, particularly within the liver, bone, and lungs. Median survival was only 3.7 months, our longest survivor died after 25 months. Presently, no patient transplanted for cholangiocellular carcinoma is alive. With few exceptions similar results have been reported by other authors.

From those disappointing results one must draw the conclusion that liver transplantation alone can not be recommended for patients with cholangiocellular carcinoma unless effective adjuvant therapy becomes available in the future. Instead, all efforts should be directed towards identification of patients with an early tumor stage who are amenable to radical surgery by partial liver resection.

In contrast, liver transplantation seems to offer some chance to patients with extrahepatic cholangiocellular tumor, the Klatskin type. If there is irresectability because of deep bilateral parenchymal infiltration or involvement of both hepatic arterial branches but no lymph node involvement, we would propose liver transplantation. Results are better than in intrahepatic cholangiocarcinoma in our series and two patients are surviving now for more than 5 years free of recurrence. Apparently there is a significant biological difference between intrahepatic and extrahepatic cholangio-carcinomas.

P. Pichlmayr
B. Ringe
Hannover, FRG.

